# Supplementary material for: Wrapping glia regulates neuronal signaling speed and precision in the peripheral nervous system of Drosophila
Source: Nat Commun. 2020 Sep 8;11:4491. doi: 10.1038/s41467-020-18291-1 (PMC7479103; doi:10.1038/s41467-020-18291-1)
Supplement: Supplementary file 1 — Supplementary information [file 41467_2020_18291_MOESM1_ESM.pdf]

Wrapping glia regulates neuronal signaling speed and precision in the peripheral nervous system of *Drosophila*

Rita Kottmeier et al.

Supplementary Table 1 Axonal diameter and wrapping index
WG> indicates *nrv2-Gal4, 90C03-Gal80*.

Genotype	# of axons	# of nerves	# of larvae	ø radius	ø wrapping index
WG>GFP	2046	27	5	0.194	18.5
WG>htl^{DN}	2555	22	5	0.159	7.5
WG>hid	1915	28	5	0.160	n.d.
WG>GFP + sphingosine	2125	26	5	0.180	19.4
WG>htl^{DN} + sphingosine	2580	31	6	0.167	14.6

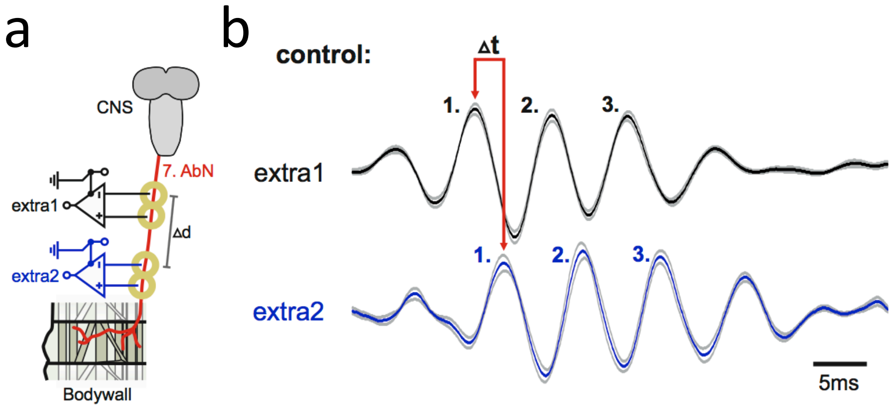

Supplementary Figure 1 Characteristic action potential trains can be recorded during fictive crawling

a During fictive crawling in an electrophysiological recording setup, characteristic trains of three action potentials can be recorded repeatedly. **b** The black line is the average of all analyzed spikes of control preparations. Grey lines indicate the standard deviation. Note that the same membrane potential pattern is recorded by the two electrodes (extra1 and extra2), which allows to calculate the time difference and thus the conduction velocity.

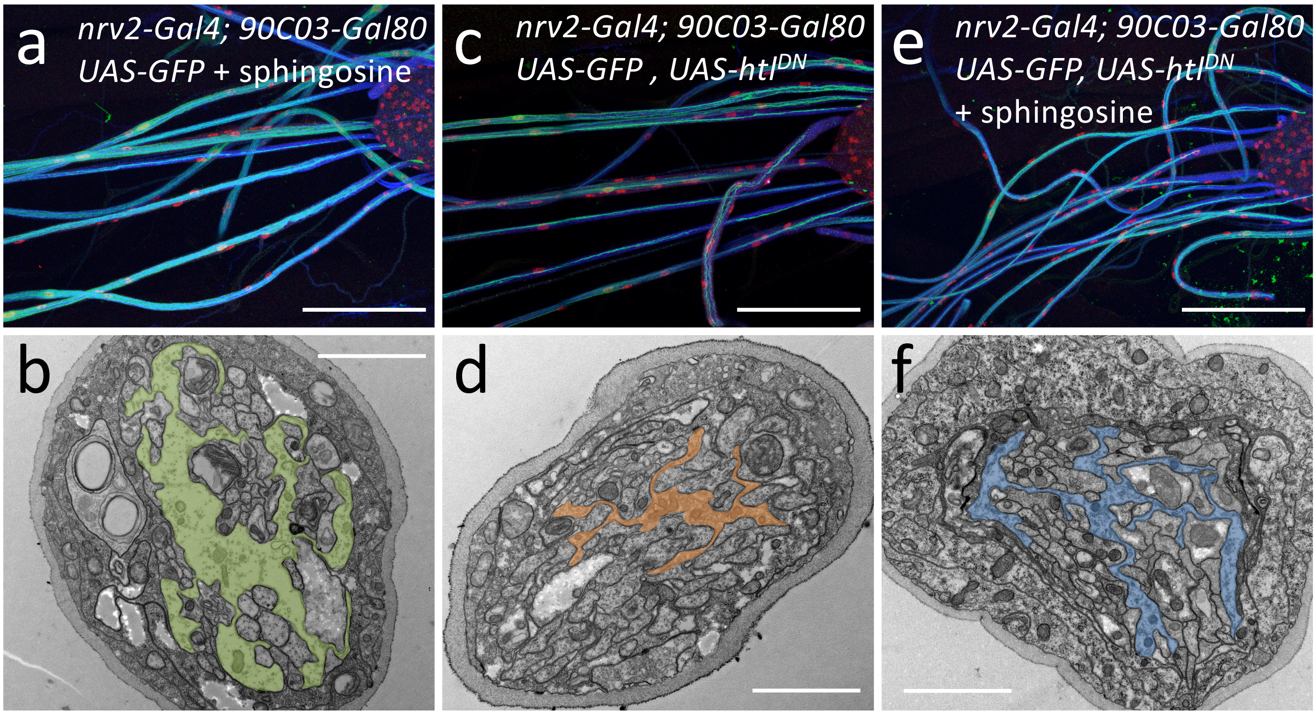

Supplementary Figure 2 Sphingosine supplement rescues glial differentiation
a,c,e Confocal images show third instar larval nervous systems stained for Repo (red), neuronal membranes (anti-HRP, blue) and wrapping glial membranes (GFP expression driven by *nrv2-Gal4 90C03-Gal80*). The different genotypes are indicated. A representative image out of six animals each is shown for every condition. Scale bars are 200 µm. **b,d,f** Corresponding electron microscopic sections taken 160 µm posterior to the tip of the ventral nerve cord. The shading indicates the wrapping glial cell shape. Five larval filets were sectioned for every genotype. A representative image is shown for every condition. Scale bars are 2 µm. **a** In control larvae fed with sphingosine, wrapping glia differentiation covers all peripheral axons. **b** In the electron microscopic image extensive ramifications of the wrapping glial cell can be detected. The wrapping glia appears slightly thicker (compare to control larvae without sphingosine see Figure 2c). **c,d** Upon expression of *htl*^{DN} in wrapping glia, the complexity of glial differentiation is reduced. **e,f** When such larvae are fed with sphingosine supplemented food, the complexity of glial differentiation increases and more glial cell processes can be detected.
