# Supplementary material for: Wrapping glia regulates neuronal signaling speed and precision in the peripheral nervous system of Drosophila
Source: Nat Commun. 2020 Sep 8;11:4491. doi: 10.1038/s41467-020-18291-1 (PMC7479103; doi:10.1038/s41467-020-18291-1)
Supplement: Supplementary file 3 — Description of Additional Supplementary Files [file 41467_2020_18291_MOESM3_ESM.pdf]

Description of Additional Supplementary Files

Title: Supplementary movie 1

Description: Locomotion of third instar control larvae.

Title: Supplementary movie 2

Description: Third instar larval locomotion upon wrapping glia ablation results in a coiling phenotype.

Title: Supplementary movie 3

Description: Third instar larval locomotion upon wrapping glia ablation results in a coiling phenotype. Close up.

Title: Supplementary movie 4

Description: Rolling behavior of third instar larvae triggered by optogenetic activation of the mdIV neurons.

Title: Supplementary movie 5

Description: Rolling behavior of third instar larvae triggered by optogenetic activation of the mdIV neurons after ablation of wrapping glial cells.

Title: Supplementary movie 6

Description: Rolling behavior of third instar larvae triggered by optogenetic activation of the Goro neurons. Larvae generally show 5 rolls in always the same orientation.

Title: Supplementary movie 7

Description: Rolling behavior of third instar larvae triggered by optogenetic activation of the Goro neurons after ablation of wrapping glial cells. Larvae show in median 9 rolls with frequent alteration in the turning orientation.
