# Supplementary material for: Wrapping glia regulates neuronal signaling speed and precision in the peripheral nervous system of Drosophila
Source: Nat Commun. 2020 Sep 8;11:4491. doi: 10.1038/s41467-020-18291-1 (PMC7479103; doi:10.1038/s41467-020-18291-1)
Supplement: Supplementary file 11 — Reporting Summary [file 41467_2020_18291_MOESM11_ESM.pdf]

nature research

Corresponding author(s): Christian Klämbt

Last updated by author(s): Jul 29, 2020

Reporting Summary

Nature Research wishes to improve the reproducibility of the work that we publish. This form provides structure for consistency and transparency in reporting. For further information on Nature Research policies, see our Editorial Policies and the Editorial Policy Checklist.

Statistics

For all statistical analyses, confirm that the following items are present in the figure legend, table legend, main text, or Methods section.

n/a	Confirmed	
☐	☒	The exact sample size (*n*) for each experimental group/condition, given as a discrete number and unit of measurement
☐	☒	A statement on whether measurements were taken from distinct samples or whether the same sample was measured repeatedly
☐	☒	The statistical test(s) used AND whether they are one- or two-sided<br>*Only common tests should be described solely by name; describe more complex techniques in the Methods section.*
☒	☐	A description of all covariates tested
☒	☐	A description of any assumptions or corrections, such as tests of normality and adjustment for multiple comparisons
☐	☒	A full description of the statistical parameters including central tendency (e.g. means) or other basic estimates (e.g. regression coefficient) AND variation (e.g. standard deviation) or associated estimates of uncertainty (e.g. confidence intervals)
☐	☒	For null hypothesis testing, the test statistic (e.g. *F*, *t*, *r*) with confidence intervals, effect sizes, degrees of freedom and *P* value noted<br>*Give P values as exact values whenever suitable.*
☒	☐	For Bayesian analysis, information on the choice of priors and Markov chain Monte Carlo settings
☒	☐	For hierarchical and complex designs, identification of the appropriate level for tests and full reporting of outcomes
☒	☐	Estimates of effect sizes (e.g. Cohen's *d*, Pearson's *r*), indicating how they were calculated

Our web collection on statistics for biologists contains articles on many of the points above.

Software and code

Policy information about availability of computer code

Data collection	FIMTrack v3.1.28.5, Zeiss imager ZEN 2.3 SP1
Data analysis	FIMAnalytics v0.1.1.2, Excel15.28, Matlab R2015a, Spike2 version 7.01; ImageJ, version 1.52b; SigmaPlot version 12

For manuscripts utilizing custom algorithms or software that are central to the research but not yet described in published literature, software must be made available to editors and reviewers. We strongly encourage code deposition in a community repository (e.g. GitHub). See the Nature Research guidelines for submitting code & software for further information.

Data

Policy information about availability of data

All manuscripts must include a data availability statement. This statement should provide the following information, where applicable:
- Accession codes, unique identifiers, or web links for publicly available datasets
- A list of figures that have associated raw data
- A description of any restrictions on data availability

All fly strains generated and the custom made scripts used in this study can be obtained through CK.

Field-specific reporting

Life sciences study design

All studies must disclose on these points even when the disclosure is negative.

Sample size	The sample size was selected based on previous experience and on the current standard in the field. For histochemical analyses more than 10 animals originating from at least three crosses were analyzed. For behavioral studies we analyzed more than 45 animals from at least three independent crosses for 180 seconds generating 1800 frames per animal. Generally highly significant p-values were obtained in statistical tests. More than five specimens were analyzed at EM level due to pratical considerations.
Data exclusions	No data exclusion for behavioral experiments. For the electrophysiological experiments outliers were determined using Grubb's test and removed.
Replication	All attemps to replicate the data were successful.
Randomization	Larvae were grouped according to their genotype, within a given genotype animals of comparable size were selected.
Blinding	All behavioral analyses were done by FIManalytics from movies taken from behaving larvae. Blinding was not done in case of electrophysiological experiments, since only one person had all the required expertise. EM sections were generated non-blinded but were analyzed blinded (by a different person). LSM analysis was performed non-blinded.

Reporting for specific materials, systems and methods

We require information from authors about some types of materials, experimental systems and methods used in many studies. Here, indicate whether each material, system or method listed is relevant to your study. If you are not sure if a list item applies to your research, read the appropriate section before selecting a response.

Materials & experimental systems

n/a	Involved in the study	
☐	☒	Antibodies
☒	☐	Eukaryotic cell lines
☒	☐	Palaeontology and archaeology
☐	☒	Animals and other organisms
☒	☐	Human research participants
☒	☐	Clinical data
☒	☐	Dual use research of concern

Methods

n/a	Involved in the study	
☒	☐	ChIP-seq
☒	☐	Flow cytometry
☒	☐	MRI-based neuroimaging

Antibodies

Antibodies used	Anti-V5 (1:500, Invitrogen, R96025); anti-Flag (1:1000; Novus biologicals, NBP1-06712); anti-HA (1:1000; Covance, MMS-101P 901503); anti-HRP-DyLightTM649 (1:500; Dianova, 123-165-021); anti-GFP (1:1000; Invitrogen, A6455); anti dsRed (1:1000, Clontech Labs 3P 632496), anti-Innexin2 (1:100, 67) anti-Futsch (1:5, mAb 22C10, 71,72), conjugated secondary antibodies (all 1:1000, anti-mouse 488, A10680; anti-mouse 568, A11031; anti-rabbit 488, A11008; anti-rabbit 568, A11011; anti guinea pig 647, 1903515; anti-rat 647, A21247; all Invitrogen).
Validation	All antibodies were validated by expression analysis. Antibodies against tags (V5, Flag, HA, dsRed, GFP) did not stain animals lacking expression of the tag, but did stain when we expressed respectively tagged proteins. Antibodies against Innexin2 or Futsch were validated using corresponding mutants. No staining is noted in mutant tissue. Information is presented in Figures 1,2,4, and 5.

Animals and other organisms

Policy information about studies involving animals; ARRIVE guidelines recommended for reporting animal research

Laboratory animals	Study involved Drosophila melanogaster. Equal numbers of female and male five days old third instar larvae were analyzed. The following fly strains were used in this study: 90C03-Gal4, MCFO-2 57,62, repo-Gal4, nrv2-Gal4, UAS-CD8Cherry, UAS-GFP, UAS-hid, R27H06-LexA (= ppk-LexA), 16E11-LexA (= Goro-LexA), LexAop-csChrimson, moody-Gal4, UAS-heartlessdsRNA, innexin2dsRNA, ogredsRNA, UAS-heartlessDN. All flies were obtained from the Bloomington or Vienna stock collections. The strain 90C03-Gal80 was generated for this study.
Wild animals	No wild animals were used in this study
Field-collected samples	No field-collected animals were used in this study
Ethics oversight	According to German and European law, work with Drosophila melanogaster does not require approval by the local Ethics committee.

Note that full information on the approval of the study protocol must also be provided in the manuscript.
